# Widely-Linear Digital Self-Interference Cancellation in Full-Duplex USRP Transceiver

**DOI:** 10.3390/s22249607

**Published:** 2022-12-08

**Authors:** Cristina Despina-Stoian, Roua Youssef, Angela Digulescu, Emanuel Radoi, Roland Gautier, Alexandru Serbanescu

**Affiliations:** 1 Lab-STICC, Université de Bretagne Occidentale, CNRS, CS 93837, 6 Avenue Le Gorgeu, CEDEX 3, 29238 Brest, France; 2 Telecommunications and Information Technology Department, Military Technical Academy “Ferdinand I”, 050141 Bucharest, Romania

**Keywords:** full-duplex communications, self-interference, I/Q imbalance, image rejection ratio, software-defined radio, widely-linear adaptive filter

## Abstract

Full-duplex (FD) communication systems allow for increased spectral efficiency but require effective self-interference cancellation (SIC) techniques to enable the proper reception of the signal of interest. The underlying idea of digital SIC is to estimate the self-interference (SI) channel based on the received signal and the known transmitted waveform. This is a challenging task since the SI channel involves, especially for mass-market FD transceivers, many nonlinear distortions produced by the impairments of the analog components from the receiving and transmitting chains. Hence, this paper first analyzes the power of the SI components under practical conditions and focuses on the most significant one, which is proven to be produced by the I/Q mixer imbalance. Then, a widely-linear digital SIC approach is adopted, which simultaneously deals with the direct SI and its image component caused by the I/Q imbalance. Finally, the performances achieved by linear and widely-linear SIC approaches are evaluated and compared using an experimental FD platform relying on software-defined radio technology and GNU Radio. Moreover, the considered experimental framework allows us to set different image rejection ratios for the transmission path I/Q mixer and to study its influence on the SIC capability of the discussed approaches.

## 1. Introduction

Full-duplex (FD) operation mode represents a new paradigm in the radio communication field, which promises a significant increase in spectral efficiency in response to the emerging problem of radio frequency (RF) spectrum overload. Involving simultaneous transmission and reception on the same RF carrier, the FD transceivers are subject to a strong self-interference (SI) signal induced in the reception path by its own transmission, which overwhelms the signal of interest (SoI).

To suppress the SI, various electromagnetic, analog, and digital self-interference cancellation (SIC) solutions have been proposed in the FD-related literature [1,2,3]. The electromagnetic SIC is performed directly on the radiated RF signal and basically consists of increasing the isolation between the receiving and transmitting antennas [4,5]. The next SIC stage acts at the analog signal processing level and makes use of the SI estimation provided by adaptive or non-adaptive multi-tap filters from the signal induced in the transmitting antenna on the uplink [6]. 

Based on the known transmitted signal, most digital SIC solutions [7,8,9,10,11] aim to eliminate the residual SI remaining after the first two cancellation stages, whose performance is limited by the analog components’ complexity and impairments. Therefore, a realistic SI model, taking into account all the important nonlinearities, is needed to improve the SIC capability of FD transceivers. The most significant sources of nonlinear distortions, including the power amplifier (PA), in-phase and quadrature (I/Q) mixer, and local oscillator (LO), have recently received increasing attention.

In order to achieve the best trade-off between cost and performance, the wireless systems adopt low-cost, direct-conversion transceivers. In addition, since the same carrier frequency is used for transmission and reception, the shared oscillator configuration is the most common for an FD transceiver. Therefore, assuming this configuration, the phase noise that occurs in the transmission path is implicitly compensated after the down-conversion so that the phase distortion can be considered negligible [10,12,13]. 

The PA nonlinear distortion has been widely studied in simulated and experimental approaches, and different estimations have been proposed [14,15,16,17]. Additionally, the I/Q imbalance has been identified as another significant distortion source engendering a strong SI image component in the SI channel of FD transceivers. According to [18,19], apart from the SI component caused by the SI channel, which can be significantly mitigated by linear SIC approaches, the SI image component represents the dominant distortion, especially for mass-market RF transceivers.

The quadrature mixer impairments are quantified by the image rejection ratio (IMRR), which represents the power ratio between the desired signal and its complex conjugate version caused by the I/Q imbalance, called image signal. Differently from the high-performance laboratory equipment that ensures an image signal attenuation of 60–80 dB, the IMRR associated with mass-market RF transceivers is typically in the range of 20–30 dB [18,20]. Consequently, especially in the case of the direct-conversion architecture of FD transceivers, the image signal results in severe distortions that can be mitigated using the conjugated transmitted signal.

The approaches that jointly consider the linear and image components of the SI signal are known as widely-linear approximations. To the best of our knowledge, the widely-linear digital SIC was first proposed in [18], where the SI signal is estimated based on the transmitted signal and its complex conjugate. 

Other recent research works aim to decrease the complexity of the widely-linear adaptive filter. In [21], the adaptive filter’s coefficients are updated based on a complex dual-channel estimation involving two independent cost functions, which correspond to the real and imaginary parts of the instantaneous error. In addition, augmented nonlinear LMS (Least Mean Squares) approaches that jointly consider the PA third-order nonlinearity and the I/Q imbalance were proposed in [11,17,19,22]. In order to minimize the SIC technique complexity, a variable periodic frequency-domain update, relying on the instantaneous square error, was introduced in [8]. 

However, most digital SIC techniques have been validated in simulated conditions, and only a few recent papers have reported results obtained using experimental setups based on the wireless open-access research platform (WARP) [23,24,25]. Since the WARP platform provides an IMRR of 40–50 dB [26,27], the effect of the I/Q imbalance can be considered negligible in all these configurations, while for the mass-market FD transceivers, the I/Q imbalance remains a serious challenge that deserves more attention. 

Hence, in this paper, we first study the I/Q imbalance influence in FD communication systems. We adopt a SIC method derived from the adaptive filtering theory that aims to minimize the conjugated SI signal due to the I/Q mixer impairments. Another important contribution of this paper consists of an experimental testbed developed using Software-Defined Radio (SDR) technology and GNU Radio (https://www.gnuradio.org/, accessed on 15 September 2022), which enables the evaluation of the proposed SIC solutions under realistic conditions. Furthermore, the Universal Software Radio Peripheral (USRP) X310 platform, which is the main element of our experimental framework, allows the software control of the IMRR value, thus being able to operate as both a mass-market and a high-performance FD transceiver. The performance of the proposed SIC algorithm is finally evaluated in terms of SIC capability in the absence of the SoI.

The remainder of the paper is organized as follows. Section 2 summarizes the main distortions due to the FD transceiver hardware impairments and studies the power levels of the introduced SI signal components. The considered linear and widely-linear SIC approaches, relying on the Recursive Least Squares (RLS) adaptive filtering algorithm, are briefly recalled in Section 3. Then, the proposed experimental setup, along with implementation details and capabilities, are specified in Section 4. Section 5 is dedicated to the performance evaluation of the proposed SIC methods, under real conditions, for different IMRR values. Finally, we present some conclusions and plan future work in Section 6.

In the following, continuous and discrete time signals are expressed in italic lowercase, while vectors are denoted by bold lowercase symbols. The operators ⊗, (·)*, and (.)*^T^* stand for signal convolution, complex conjugate, and vector transposition, respectively. The acronyms used throughout the paper are also summarized in Table 1.

## 2. Self-Interference in Full-Duplex Transceivers

**Figure 1:**
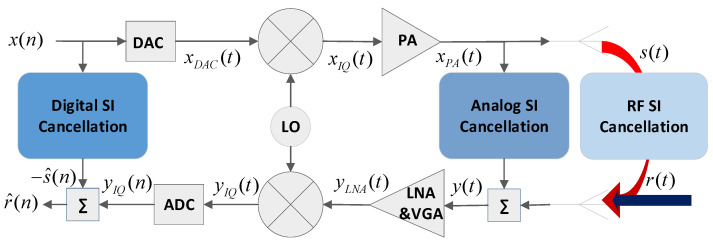
FD transceiver block diagram and associated signals.

The block diagram of a direct-conversion FD transceiver is depicted in Figure 1, along with the main continuous and discrete time signals required for describing its operating principle. The SI, denoted by *s*(*t*), is a part of the power amplifier’s (PA) output signal $x_{PA}\left(t\right)\;$, which reaches the receiving antenna through an SI channel $h_{SI}\left(t\right)$ encompassing the RF cancellation stage.

As expressed in Equation (1), the received signal *y*(*t*) is the superposition of the SoI *r*(*t*), which the FD system aims to receive from the correspondent transceiver, the SI signal *s*(*t*) induced by the transceiver’s own transmission, and an additive white Gaussian noise (AWGN) *z*(*t*).

(1) $$y(t)=h_{SI}(t)⊗x_{PA}(t)+r(t)+z(t).$$

In addition to the SI channel effects, the SI signal includes nonlinear distortions due to the impairments of the analog components involved in the transmission and reception chains. According to the FD-related literature, the transmitter I/Q imbalance and the PA nonlinear effects dominate the residual SI [18,19,28].

### 2.1. I/Q Mixer Imbalance

The quadrature mixer represents a significant source of nonlinearities, especially in direct-conversion transceivers. The amplitude and phase mismatches between the parallel channels dealing with the I and Q components, known as I/Q imbalance, result in an image signal that corresponds to the complex conjugate of the original signal. According to Figure 1 and assuming a frequency-dependent model for the up-conversion transformation on the transmission path, the signal distorted by I/Q imbalance can be expressed as:

(2) $$\;\;x_{IQ}(t)=g_{1}^{Tx}(t)⊗x(t)+g_{2}^{Tx}(t)⊗x_{}^{*}(t),$$

with:

(3) $$\;g_{1}^{Tx}(t)=\frac{g_{I}^{Tx}(t)+\gamma g_{Q}^{Tx}(t)e^{-j\varphi }}{2},\;g_{2}^{Tx}(t)=\frac{g_{I}^{Tx}(t)-\gamma g_{Q}^{Tx}(t)e^{j\varphi }}{2},$$

where $\left\{g_{I}^{Tx}(t),\;g_{Q}^{Tx}(t)\right\}$ represent the impulse responses of low-pass filters for I and Q channels, respectively, including the amplitude (γ) and phase (φ) mismatches [29].

The I/Q imbalance is characterized by the power ratio between the signal and its image component, known as the image rejection rate, and calculated as:

(4) $$\;IMRR(f)=\frac{Ε\left[{\left|X(f)G_{1}^{Tx}(f)\right|}^{2}\right]}{Ε\left[{\left|X^{*}(-f)G_{2}^{Tx}(f)\right|}^{2}\right]}=\frac{Ε\left[{\left|G_{1}^{Tx}(f)\right|}^{2}\right]}{Ε\left[{\left|G_{2}^{Tx}(f)\right|}^{2}\right]},$$

where $G_{1}^{Tx}(f)$ and $G_{2}^{Tx}(f)$ stand for the transfer functions corresponding to $g_{1}^{Tx}(t)$ and $g_{2}^{Tx}(t)$, respectively [30].

In the same way, at the reception path, the output of the receiver’s I/Q mixer can be expressed as:

(5) $$\;\;y_{IQ}(t)=g_{1}^{Rx}(t)⊗y_{LNA}(t)+g_{2}^{Rx}(t)⊗y_{LNA}^{*}(t),$$

where $\left\{g_{1}^{Rx}(t),\;g_{2}^{Rx}(t)\right\}$ are defined similarly to $\left\{g_{1}^{Tx}(t),\;g_{2}^{Tx}(t)\right\}$, using the impulse responses of the down-conversion related low-pass filters, while $\;\;y_{LNA}(t)$ is the output signal of the considered ideal LNA, providing an amplification factor of $k_{LNA}$:

(6) $$\;\;y_{LNA}(t)=k_{LNA}y(t).$$

### 2.2. Power Amplifier Nonlinearity

For energy-efficient communications systems, the PA typically operates close to its saturation point, leading to significant nonlinear distortions and short-term memory effects. Thus, the PA nonlinear behavior results in spectral regrowth and new harmonic components at multiples of the carrier frequency.

The PA nonlinear behavior and its estimation have been widely studied by both simulated and experimental approaches. Recently, increasingly accurate models have been developed based on the Volterra series and involving Taylor series expansion to estimate the PA nonlinear distortions [14,15,16].

In order to decrease the model complexity and the number of parameters that need to be estimated, other derived models, such as Weiner or Hammerstein, which can be used in different parallel or serial coin terms of complexity and accuracy, are provided in [31], which concludes that for an AB class power amplifier, the parallel Hammerstein (PH) model provides the most accurate estimation involving a low number of parameters. This characteristic makes it particularly suitable for SI cancellation approaches [17,32,33].

According to this approximation model, the PA output signal can be expressed as:

(7) $$\;x_{PA}(t)=\sum_{p=0}^{P}\sum_{n=0}^{N_{PH}}a_{2p+1}^{}(n)x_{IQ}(t-nT_{s}){\left|x_{IQ}(t-nT_{s})\right|}^{2p},$$

where *N_PH_* is the memory length of the PH model with nonlinearity order (2*P* + 1), $T_{s}$ stands for the sampling period, $a_{2p+1}(n)$ is the equivalent filter impulse response for the (2*p +* 1)*^th^* order harmonic, and $x_{IQ}(t)$ denotes the signal at the output of the I/Q transmitter’s mixer. For simplicity, only the third-order nonlinear component (IMD), further denoted as $x_{IMD}(t)$, is considered, so that the PA output signal is given by:

(8) $$\;x_{PA}(t)=\sum_{n=0}^{N_{PH}}a_{0}^{}(n)x_{IQ}(t-nT_{s})+\underset{x_{IMD}(t)}{\underbrace{\sum_{n=0}^{N_{PH}}a_{3}^{}(n)x_{IQ}(t-nT_{s}){\left|x_{IQ}(t-nT_{s})\right|}^{2}}},$$

### 2.3. Power of SI Components

Relying on the SI components discussed above, we can now express the discrete-time SI signal *s*(*n*) at the output of the ADC. To this aim, let us consider the impulse responses of the channels that encompass all the effects affecting the main SI components, including the SI mitigation in the analog and RF domains, as in [18]. They are denoted $h_{lin}$ and $h_{lin,conj}$ for the SI channels seen by the FD transceiver’s own transmitted signal *x*(*n*) and its conjugate $x^{*}(n)$, respectively. Similarly, $h_{IMD}$ and $h_{IMD,conj}$ correspond to the SI channels seen by the third-order nonlinear component of the PA output signal $x_{IMD}(n)$ and its conjugate $x_{IMD}^{*}(n)$, respectively.

The discrete-time SI signal is then obtained as the sum of the convolution products between the four signals mentioned above and the associated impulse responses of the corresponding SI channels:

(9) $$\begin{array}{ll} \;s(n) & =h_{lin}^{}(n)⊗x(n)+h_{lin,conj}^{}(n)⊗x^{*}(n)+h_{IMD}^{}(n)⊗x_{IMD}(n)\\ & {{}^{}}^{}{{}_{}}_{}{{}_{}}_{}{}^{{}_{}}+h_{IMD,conj}^{}(n)⊗x_{IMD}^{*}(n)+q(n)+z(n). \end{array}$$

As can be seen from (9), in addition to the distortions introduced by the PA nonlinearity and I/Q imbalance, the SI signal *s*(*n*) also includes the quantization noise *q*(*n*) and the thermal noise *z*(*n*). According to [11,18,22], *z*(*n*) is an AWGN, and its variance is computed as in (10):

(10) $$\sigma_{z}^{2}=\frac{k_{BB}k_{LNA}k_{IQ}p_{sen}}{SNR_{req}}.$$

The quantization noise *q*(*n*) is also considered to be an AWGN [34,35] characterized by its variance given by (11):

(11) $$\sigma_{q}^{2}=\frac{p_{ADC}}{10^{6.02b+4.76-PAPR/10}}.$$

The physical meaning of the parameters involved in Equations (10) and (11) are summarized in Table 2.

Based on the suggestions presented in [11,18], the power levels of the SI components are studied considering two typical direct-conversion FD transceivers, whose main parameters are listed in the table above. In accordance with the 3GPP LTE specifications [18,20], an IMRR value of 25 dB is chosen for the *Type 1* transceiver, which is typical for mass-market RF transceivers, while an IMRR value of 40 dB, specific for high-tech communications systems and widely used during laboratory validation tests, is taken for the *Type 2* FD transceiver. With the exception of the I/Q mixer performance, all the other operating parameters are identical in both considered scenarios.

**Figure 2:**
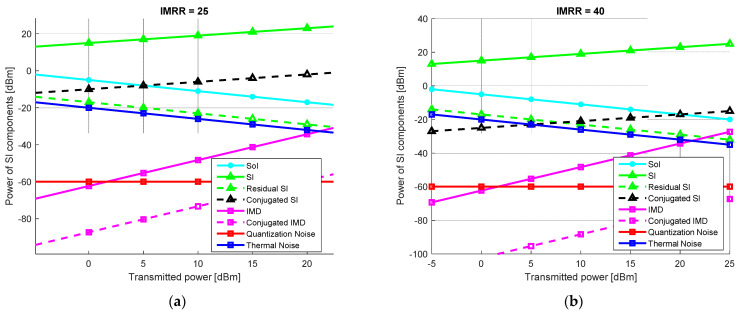
Power of SI components w.r.t. the transmitted power of an FD transceiver of (**a**) *Type 1*; (**b**) *Type 2*.

The SI signal is investigated assuming an FD transceiver with RF and analog SI cancellation capabilities of 40 dB and 30 dB, respectively. Moreover, to highlight the influence of the distortions over the SoI transmitted by the corresponding transceiver, its power at the FD receiver’s input is set at 15 dB above the thermal noise. Assuming the FD transceiver’s parameters listed in Table 2, the power levels of the SI components induced in the reception path for mass-market (*Type 1*) and high-performance (*Type 2*) FD transceivers are analyzed comparatively by simulation. The results, shown in Figure 2, are achieved considering a transmission power of the FD transceiver ranging from −5 dBm to 25 dBm. 

It can be noticed that for both FD systems, the dominant SI components are represented by the linear SI (*SI*) and the conjugated SI signal (*Conjugated SI*). Moreover, considering a linear digital approach, with 27–57 dB SI cancellation, which is feasible under realistic conditions [11,18,36,37] depending on the transmit power, the linear SI component can be further attenuated. Consequently, the residual SI level (*Residual SI*) achieved after the linear SIC is below that of the SoI and comparable to the thermal noise.

Assuming only the linear SI cancellation solution is implemented, it is clear that the distortion produced by the I/Q mixer imbalance still seriously degrades the signal-to-interference and noise ratio (SINR) of the FD transceiver. Indeed, the power of the conjugated SI exceeds that of the SoI, making it impossible to perform a coherent reception, especially in the case of higher transmitted power. 

Note that for the *Type 1* FD transceiver, this is true over almost the whole range of the considered transmitted power. Even if it is not such a critical problem in the case of a *Type 2* FD system, when the transmitted power is above 20 dBm, the conjugated SI is still more powerful than the SoI. Therefore, regardless of the FD transceiver performance, the development of SIC solutions taking into account the I/Q mixer imbalance effect is mandatory.

## 3. Adaptive Filtering for Self-Interference Cancellation

The digital SIC approaches derived from the adaptive filtering theory aim to achieve an accurate estimation of the SI signal based on the known baseband transmitted signal. The adaptive filter is designed to regenerate the SI signal, including the SI channel effects, in order to subtract it from the received signal and thus achieve the SIC. 

Under practical conditions, the SIC capability of the adaptive filter is limited by the presence of thermal noise and nonlinear distortions introduced by the impairments of the analog Tx and Rx components. The SIC performance is strongly related to the SI approximation accuracy. It is typically evaluated, in the absence of the SoI, as the power ratio between the SI signal and the residual SI signal:

(12) $$SIC_{dB}=10\log_{10}\left(\frac{{\sum_{n}\left|s(n)\right|}^{2}}{{\sum_{n}\left|s(n)-\hat{s}(n)\right|}^{2}}\right).$$

### 3.1. Linear Self-Interference Cancellation

The adaptive filter performs a linear SIC and ignores any non-linear signal distortion. It regenerates the SI signal according to the equation below:

(13) $$\;{\hat{s}}_{lin}(n)=h^{T}(n)x(n),$$

where h(n)∈ℂ represents the (*N* × 1) vector of the adaptive filter’s coefficients that models the SI channel, including the memory effect, while x(n) is a (*N* × 1) vector formed by *N* consecutive samples of the baseband transmitted signal. 

### 3.2. Widely-Linear Self-Interference Cancellation

According to the widely-linear approach [18], the SI signal can be estimated based on the transmitted signal and its conjugate version according to Equation (14):

(14) $$\;{\hat{s}}_{wlin}(n)=h_{1}^{T}(n)x(n)+h_{2}^{T}(n)x^{*}(n),$$

where $\left\{h_{1}(n),\;h_{2}(n)\right\}\in \mathbb{C}$ represent the (*N* × 1) vectors of filter’s coefficients modeling the linear and image channel responses. Furthermore, Equation (14) may be collapsed and expressed as in Equation (15):

(15) $$\;{\hat{s}}_{wlin}(n)=\left[h_{1}^{T}(n),h_{2}^{T}(n)\right]\left[\begin{array}{l} x(n)\\ x^{*}(n) \end{array}\right]=h_{wlin}^{}(n)\left[\begin{array}{l} x(n)\\ x^{*}(n) \end{array}\right],$$

where the (1 × 2*N*) vector $h_{wlin}(n)$ simultaneously models the linear and conjugated channel effects.

The adaptive filter’s coefficients can then be calculated relying on the Wiener solution or using its different iterative approximations provided by the Least Mean Squares (LMS), Normalized Least Mean Squares (NLMS), or Recursive Least Squares (RLS) algorithms. By comparing these different algorithms, we concluded that the RLS achieves the best performance in terms of estimation accuracy, stability, and convergence speed [9].

## 4. USRP-Based Experimental Setup

Due to the SI channel complexity, it is extremely challenging to perform a realistic simulation of the operation mode of FD communication systems. Thus, it is much more convenient to develop experimental testbeds for the validation and evaluation of the proposed SIC approaches. USRP-based platforms [7] embedding SDR technology represent promising tools for testing and evaluating the performance of state-of-the-art solutions in the radio communications domain.

### 4.1. USRP Framework

**Figure 3:**
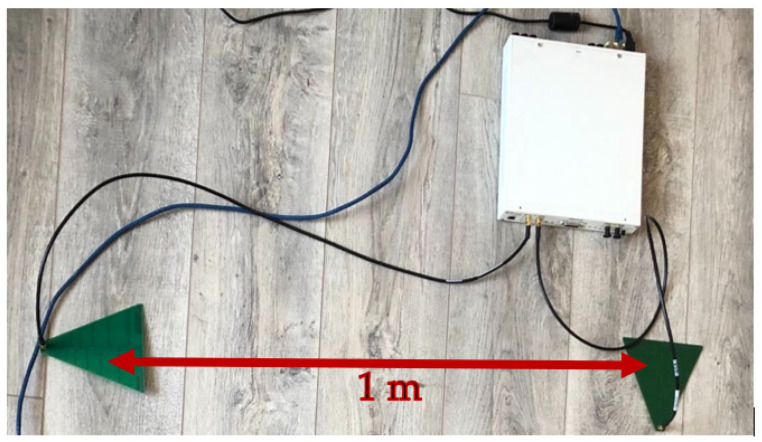
Experimental setup for FD transceiver testing.

The experimental testbed, shown in Figure 3, consists of a USRP X310 platform that operates in FD mode and is driven by GNU Radio via Universal Hardware Driver (UHD). The USRP X310 platform consists of a UBX 160 USRP daughterboard that covers frequencies from 10 MHz to 6 GHz with up to 160 MHz of instantaneous bandwidth [38]. GNU Radio allows controlling the USRP’s transmission power by a gain control tunable parameter, whose values vary from 0 to 31.5 dB for the USRP X310 platform. Thus, the SI signal power can be varied in quite a wide range [39].

The FD transceiver, relying on the USRP X310 platform, incorporates log periodic antennas LP0965 operating in the 850 MHz–6.5 GHz frequency range and having a gain of 5–6 dBi [40]. As can be noticed in Figure 3, the reception and transmission antennas are arranged out of phase with 90°, at a distance of 1 m to ensure an additional SI cancellation in the RF field that prevents the ADC saturation.

The FD transceiver involves a residual SI signal that can be characterized, in the digital domain, by the self-interference to noise power ratio (INR). In our framework, under a fixed receiver gain of 5 dB, the INR can be varied linearly, depending on the gain defined on the transmission path, between 14 and 44 dB relative to a −47.65 dBm noise floor, measured when no transmission occurs. Hence, we select three representative INR values, summarized in Table 3, that are further used in the next section to assess the FD transceiver performance.

### 4.2. Software-Based Control of I/Q Impairments

The USRP X310 platform allows introducing a software-controlled I/Q imbalance using Ettus’ UHD utility *set_iq_imbalance*, which is integrated as a function in GNU Radio companion. The complex correction factor is applied to the RF daughterboard transmit path. To evaluate the IMRR as a function of the correction factor variation, a single frequency tone is transmitted, and the power levels of the desired and image components are measured on the reception path.

**Figure 4:**
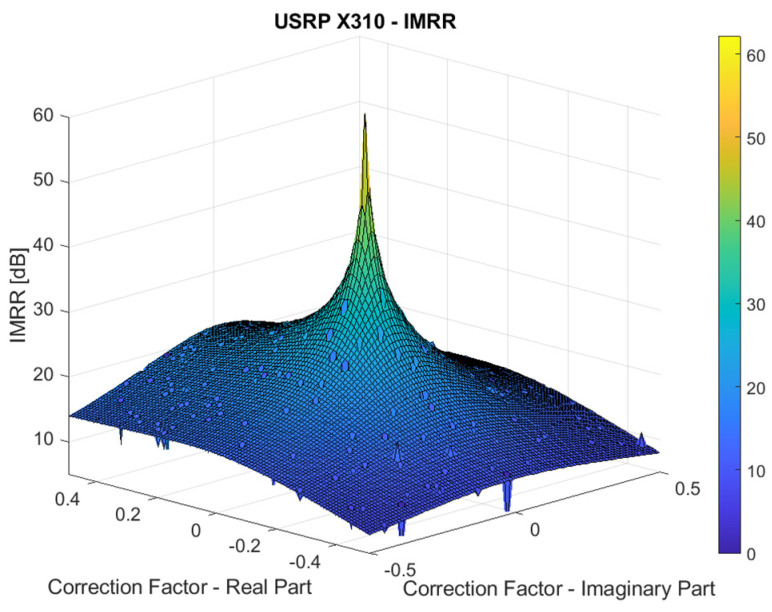
IMRR variation with respect to the correction factor for USRP X310 platform.

The IMRR results, given in Figure 4, are measured at a center frequency of 2.5 GHz for real and imaginary parts of the correction factor individually swept between −0.5 and 0.5. It can be noticed that the UBX daughterboard enables a quite wide range of IMRR values, from 0 to almost 50 dB, and allows evaluating and validating our SIC algorithms, under realistic conditions, for both mass-market and high-performance operation modes of FD transceivers. Hence, we selected four representative IMRR values and the corresponding correction factors, summarized in Table 4, which are further used in the next section to assess the FD transceiver performance.

## 5. Measurements and Results

The adaptive filtering SIC approaches presented in Section 2 have been evaluated using the experimental setup described in Section 4 in terms of SIC capability expressed in Equation (12). Each transmitted baseband SI frame consists of a PN11 pseudorandom sequence of 2047 bits, followed by a random sequence of 10^5^ bits. In our experimental testbed, 10 frames of the SI signal with this structure are generated using the QPSK modulation.

All the measurements were performed by setting the RF carrier frequency to 2.5 GHz and the sampling rate to 5 MHz. The SIC capability achieved by the linear and widely-linear adaptive filtering approaches was evaluated according to Equation (12) for the different INR and IMRR values summarized in Table 3 and Table 4, respectively. The chosen IMRR values correspond to transceivers with high (IMRR = 40 dB, IMRR = 35 dB), medium (IMRR = 30 dB), and low performance (IMRR = 25 dB).

For both adaptive approaches, the filter’s coefficients are continuously updated based on the RLS algorithm. For each configuration, the linear and widely-linear SIC approaches are evaluated for a number of coefficients ranging from 50 to 300, with a step size of 50 coefficients. 

**Figure 5:**
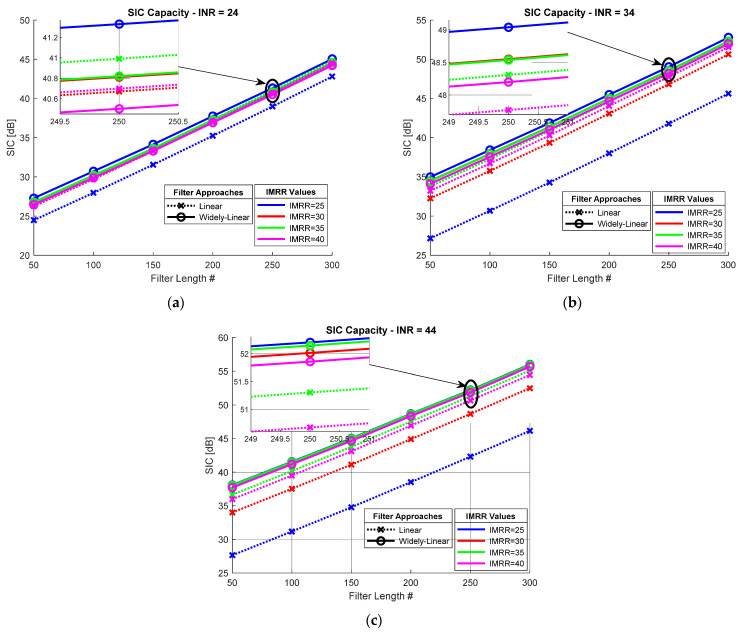
Digital SIC capability achieved for different IMRR values considering: (**a**) INR = 24 dB, (**b**) INR = 34 dB, and (**c**) INR = 44 dB.

Figure 5 shows the SIC capability achieved considering different levels of performance for the I/Q mixer. Note that the SIC capability of both approaches increases linearly with the adaptive filter length, while the gaps between the SIC capabilities associated with the various configurations remain constant. It can also be noticed that in most configurations, the widely-linear adaptive filtering approach outperforms the linear one. 

There are only two exceptions, shown in Figure 5a, which correspond to an INR of 24 dB, i.e., limited transmission power of the FD transceiver. Indeed, in this case, the linear approach achieves a higher SIC capability than the widely-linear one for IMRR values of 35 and 40 dB (high-performance transceivers). 

This behavior is due to the reduced power of the SI conjugate component, caused by the I/Q imbalance, which is negligible compared to the linear SI component for low transmission powers, according to the observations presented in Section 2.3. In this case, the SIC capabilities obtained by the widely-linear SIC method are slightly reduced because half of the filter coefficients aim at estimating the conjugated SI component, thus limiting the accuracy of the linear SI component approximation. However, the results obtained by the two approaches remain close, the SIC capability gap being only 0.2 dB and 0.3 dB, for an IMRR of 40 dB and 35 dB, respectively.

Additionally, in the case of medium-performance transceivers, characterized by an IMRR of about 30 dB, the widely-linear approach slightly outperforms the linear one, with an increase of about 0.2 dB in SIC capability. The only significant improvement of about 2 dB, provided by the widely-linear SIC method, is achieved in the case of mass-market transceivers with limited performance, characterized by an IMRR of 25 dB. 

Nevertheless, according to the results shown in Figure 5b,c, for higher transmission powers, represented by INR values of 34 dB and 44 dB, the widely-linear method provides better SIC capabilities regardless of the I/Q mixer performance. Furthermore, for a given INR, the widely-linear technique performs similarly for all the considered IMMRs; the SIC capability variation is less than 0.8 dB and 0.5 dB, respectively, for these two INR values, while it is about 6 dB and 9 dB, respectively, for the linear SIC technique. We can therefore conclude that the widely-linear SIC method removes the conjugated SI component in a fairly efficient manner regardless of its strength, while the linear approach drastically degrades its cancellation performance as the strength of this component grows. 

As expected from the observations reported in Section 2.3, compared to the linear approach, the widely-linear approach offers the most notable gains in SIC capability (7 dB and 12 dB for INR values of 34 dB and 44 dB, respectively) in the case of mass-marker FD transceivers (IMRR = 25 dB). Moderate gains (almost 4 dB and 2 dB for INR values of 44 dB and 34 dB, respectively) are also achieved for medium-performance FD transceivers (IMRR = 30 dB), while the SIC capability gain is much more reduced in the case of high-performance FD communication systems. 

Overall, the results presented in Figure 5 suggest that the widely-linear approach outperforms the linear one in terms of SIC capability, especially in the case of mass-market communication systems transmitting high power. In addition, the widely-linear SIC decreases the computational burden associated with the cancellation method by reducing the number of coefficients of the adaptive filter. Indeed, according to Figure 5c, 300 coefficients are required for a linear adaptive filter to achieve a SIC capability of 47 dB so as to reduce the SI signal below the noise level in the case of mass-market FD transceivers (IMRR = 25 dB). In order to reach the same goal, the widely-linear approach only needs 150 coefficients, which results in decreasing the computational cost by half.

## 6. Conclusions and Future Work

Digital SIC techniques derived from adaptive filtering solutions represent a promising solution in the FD communication field. Considering the huge complexity of any analytical model associated with realistic FD simulations, experimental validation is a highly attractive alternative for the performance evaluation of the proposed SIC techniques.

Therefore, this paper analyzes the power of the SI components due to the SI channel and analog component impairments involved in FD communications systems, considering different performance parameters. After establishing that linear and conjugated SI, caused by the mixer I/Q imbalance, are the dominant SI components, we evaluate the improvement in digital SIC capability achieved by an adaptive filtering-based widely-linear approach.

The effectiveness of adaptive SIC solutions strongly depends on the FD transceiver performance. Hence, the main contribution of this paper is represented by the development of a testbed platform using USRP X310 SDR modules, which allows evaluating the SIC effectiveness under realistic conditions and considering different performance parameters of the FD transceiver. Our work focuses on the analysis of the distortion introduced by the I/Q mixer, whose performance is characterized using the IMRR. 

Thus, the linear and widely-linear approaches are evaluated in terms of SIC capability, considering low, medium, and high-performance FD transceivers. It has been observed that the widely-linear approach provides a significant improvement in terms of SIC capability, especially in the case of mass-market FD communication systems, while for high-performance FD transceivers, the SIC capabilities achieved by both approaches are rather similar. 

There are still many challenging aspects related to FD communications that need to be further investigated. In our upcoming work, we intend to improve the performance of the proposed digital cancellation stage by incorporating SIC techniques relying on neural networks. We also plan to develop our experimental framework, extended with RF and analog SIC stages, and to evaluate the proposed digital SIC techniques in terms of both SIC capability and BER. 

## Figures and Tables

**Table 1:** Acronyms used throughout the paper.

Acronym	Signification
ADC	Analog to Digital Converter
AWGN	Zero-mean Additive White Gaussian Noise
DAC	Digital to Analog Converter
FD	Full-Duplex
IMD	Third-order Nonlinear Component
IMRR	Image Rejection Rate
INR	Interference to Noise Ratio
LNA	Low Noise Amplifier
LO	Local Oscillator
PA	Power Amplifier
RF	Radio Frequency
RLS	Recursive Least Squares
Rx	Receiver
SDR	Software-Defined Radio
SI	Self-Interference
SIC	Self-Interference Cancellation
SINR	Signal-to-Interference and Noise Ratio
SoI	Signal of Interest
Tx	Transmitter
UHD	Universal Hardware Driver
USRP	Universal Software Radio Peripheral
VGA	Variable Gain Amplifier

**Table 2:** Typical parameters for FD transceivers and their notations.

Parameter	Symbol	Value
Receiver sensitivity	$\;p_{sen}$	−89 dBm
SNR requirement	$\;SNR_{req}$	15 dB
Transmit power	$\;p_{x}$	−5–25 dBm
RF cancellation	$\;a_{RF}$	40 dB
Analog cancellation	$\;a_{anl}$	30 dB
PA gain	$\;a_{0}$	27 dB
PA IMD attenuation	$\;a_{1}$	−80 dB
Rx VGA gain	$\;k_{BB}$	1–51 dB
LNA gain	$\;k_{LNA}$	25 dB
Tx and Rx mixer gain	$\;k_{IQ}$	6 dB
IMRR	*-*	25 dB (*Type 1*)
		40 dB (*Type 2*)
ADC dynamic range	$\;p_{ADC}$	7 dB
Peak-to-average-power ratio	*PAPR*	10 dB
ADC bits	*b*	12

**Table 3:** Considered transmission gains and the corresponding measured INR values.

Transmission Gain [dB]	INR Values [dB]
10	24
20	34
30	44

**Table 4:** Considered IMRR values and the corresponding correction factors.

IMRR [dB]	Correction Factor
25	0.098 − j 0.953
30	0.008 − j 0.051
35	0.015 − j 0.015
40	−0.021 + j 0.018

## Data Availability

The data presented in this study are available upon request from the corresponding author.

## References

[1] Liu G., Yu F.R., Ji H., Leung V.C.M., Li X. (2015). In-Band Full-Duplex Relaying: A Survey, Research Issues and Challenges. IEEE Commun. Surv. Tutor..

[2] Kim D., Lee H., Hong D. (2015). A Survey of In-Band Full-Duplex Transmission: From the Perspective of PHY and MAC Layers. IEEE Commun. Surv. Tutor..

[3] Zhang Z., Long K., Vasilakos A.V., Hanzo L. Full-Duplex Wireless Communications: Challenges, Solutions, and Future Research Directions. Proceedings of the IEEE 2016.

[4] Korpi D., Heino M., Icheln C., Haneda K., Valkama M. (2017). Compact Inband Full-Duplex Relays With Beyond 100 dB Self-Interference Suppression: Enabling Techniques and Field Measurements. IEEE Trans. Antennas Propagat..

[5] Everett E., Sahai A., Sabharwal A. (2014). Passive Self-Interference Suppression for Full-Duplex Infrastructure Nodes. IEEE Trans. Wirel. Commun..

[6] Huang X., Guo Y.J. (2017). Radio Frequency Self-Interference Cancellation with Analog Least Mean-Square Loop. IEEE Trans. Microw. Theory Techn..

[7] Despina-Stoian C., Youssef R., Digulescu-Popescu A., Radoi E., Alexandra S. USRP Experimental Approach for Digital Self-Interference Cancellation in Full-Duplex Communications. Proceedings of the International Conference on Advanced Technologies for Communications (ATC2021).

[8] Zhang S., Zhang J., Xia Y., So H.C. (2021). Adaptive Frequency-Domain Normalized Implementations of Widely-Linear Complex-Valued Filter. IEEE Trans. Signal Process..

[9] Despina-Stoian C., Digulescu-Popescu A., Alexandra S., Youssef R., Radoi E. Comparison of Adaptive Filtering Strategies for Self-Interference Cancellation in LTE Communication Systems. Proceedings of the 13th International Conference on Communications.

[10] Quan X., Liu Y., Shao S., Huang C., Tang Y. (2017). Impacts of Phase Noise on Digital Self-Interference Cancellation in Full-Duplex Communications. IEEE Trans. Signal Process..

[11] Li Z., Xia Y., Pei W., Wang K., Mandic D.P. (2018). An Augmented Nonlinear LMS for Digital Self-Interference Cancellation in Full-Duplex Direct-Conversion Transceivers. IEEE Trans. Signal Process..

[12] Sahai A., Patel G., Dick C., Sabharwal A. (2013). On the Impact of Phase Noise on Active Cancelation in Wireless Full-Duplex. IEEE Trans. Veh. Technol..

[13] Korpi D. (2017). Full-Duplex Wireless: Self-interference Modeling, Digital Cancellation, and System Studies. Ph.D. Thesis.

[14] Feng L. (2012). Linearization of Power Amplifiers in Wide Band Communication Systems by Digital Baseband Predistortion Technique. Ph.D. Thesis.

[15] Galaviz-Aguilar J.A., Vargas-Rosales C., Cárdenas-Valdez J.R., Martínez-Reyes Y., Inzunza-González E., Sandoval-Ibarra Y., Núñez-Pérez J.C. (2021). A Weighted Linearization Method for Highly RF-PA Nonlinear Behavior Based on the Compression Region Identification. Appl. Sci..

[16] O’Droma M., Meza S., Lei Y. (2009). New modified saleh models for memoryless nonlinear power amplifier behavioural modelling. IEEE Commun. Lett..

[17] Balatsoukas-Stimming A. Non-Linear Digital Self-Interference Cancellation for In-Band Full-Duplex Radios Using Neural Networks. Proceedings of the 2018 IEEE 19th International Workshop on Signal Processing Advances in Wireless Communications (SPAWC).

[18] Korpi D., Anttila L., Syrjala V., Valkama M. (2014). Widely linear digital self-interference cancellation in direct-conversion full-duplex transceiver. IEEE J. Select. Areas Commun..

[19] Komatsu K., Miyaji Y., Uehara H. (2020). Iterative Nonlinear Self-Interference Cancellation for In-Band Full-Duplex Wireless Communications under Mixer Imbalance and Amplifier Nonlinearity. IEEE Trans. Wirel. Commun..

[20] Li Z., Xia Y., Pei W., Mandic D.P. (2019). A cost-effective nonlinear self-interference canceller in full-duplex direct-conversion transceivers. Signal Process..

[21] Sankhe K., Belgiovine M., Zhou F., Riyaz S., Ioannidis S., Chowdhury K. ORACLE: Optimized Radio clAssification through Convolutional neuraL nEtworks. Proceedings of the IEEE INFOCOM 2019-IEEE Conference on Computer Communications.

[22] Korpi D., Anttila L., Valkama M. (2017). Nonlinear self-interference cancellation in MIMO full-duplex transceivers under crosstalk. J. Wirel. Com. Netw..

[23] Duarte M., Dick C., Sabharwal A. (2012). Experiment-Driven Characterization of Full-Duplex Wireless Systems. IEEE Trans. Wirel. Commun..

[24] Duarte M., Sabharwal A. Full-duplex wireless communications using off-the-shelf radios: Feasibility and first results. Proceedings of the 2010 Conference Record of the Forty Fourth Asilomar Conference on Signals, Systems and Computers.

[25] Jain M., Choi J., Kim T., Bharadia D., Seth S., Srinivasan K., Levis P., Katti S., Sinha P. Practical, real-time, full duplex wireless. Proceedings of the 17th annual international conference on Mobile computing and networking-MobiCom ’11.

[26] Maxim Integrated, ‘MAX2828: Single-/Dual-Band 802.11a/b/g World-Band Transceiver ICs’. https://www.maximintegrated.com/en/products/comms/wireless-rf/MAX2828.html.

[27] Maxim Integrated, ‘MAX2829: Single-/Dual-Band 802.11a/b/g World-Band Transceiver ICs’. https://www.maximintegrated.com/en/products/comms/wireless-rf/MAX2829.html.

[28] Syrjala V., Valkama M., Anttila L., Riihonen T., Korpi D. (2014). Analysis of Oscillator Phase-Noise Effects on Self-Interference Cancellation in Full-Duplex OFDM Radio Transceivers. IEEE Trans. Wirel. Commun..

[29] Tsai Y., Yen C.-P., Wang X. (2010). Blind Frequency-Dependent I/Q Imbalance Compensation for Direct-Conversion Receivers. IEEE Trans. Wirel. Commun..

[30] Peng X., Wang Z., Mo J., Wang C., Liu J., Yu F. (2020). A Blind Calibration Model for I/Q Imbalances of Wideband Zero-IF Receivers. Electronics.

[31] Tehrani A.S., Cao H., Afsardoost S., Eriksson T., Isaksson M., Fager C. (2010). A Comparative Analysis of the Complexity/Accuracy Tradeoff in Power Amplifier Behavioral Models. IEEE Trans. Microw. Theory Techn..

[32] Schoukens M., Tiels K. (2017). Identification of block-oriented nonlinear systems starting from linear approximations: A survey. Automatica.

[33] Kiayani A., Waheed M.Z., Anttila L., Abdelaziz M., Korpi D., Syrjälä V., Kosunen M., Stadius K., Ryynänen J., Valkama M. (2018). Adaptive Nonlinear RF Cancellation for Improved Isolation in Simultaneous Transmit–Receive Systems. IEEE Trans. Microw. Theory Techn..

[34] Korpi D., Riihonen T., Syrjälä V., Anttila L., Valkama M., Wichman R. (2014). Full-Duplex Transceiver System Calculations: Analysis of ADC and Linearity Challenges. IEEE Trans. Wirel. Commun..

[35] Ordonez L.G., Ferrand P., Duarte M., Guillaud M., Yang G. (2021). On Full-Duplex Radios with Modulo-ADCs. IEEE Open J. Commun. Soc..

[36] Ahmed E., Eltawil A.M. (2015). All-Digital Self-Interference Cancellation Technique for Full-Duplex Systems. IEEE Trans. Wirel. Commun..

[37] Vuong B.Q., Gautier R., Ta H.Q., Nguyen L.L., Fiche A., Marazin M. (2022). Joint Semi-Blind Self-Interference Cancellation and Equalisation Processes in 5G QC-LDPC-Encoded Short-Packet Full-Duplex Transmissions. Sensors.

[38] UBX 10-6000 MHz Rx/Tx. https://www.ettus.com/all-products/ubx160/.

[39] RF Characterization Data UBS USRP Daughterboard. https://files.ettus.com/performance_data/ubx/UBX-without-UHD-corrections.pdf.

[40] LP0965 Antenna. https://www.ettus.com/all-products/lp0965/.

